# Housestaff perceptions on training and discussing the Maryland Orders for Life Sustaining Treatment Form (MOLST)

**DOI:** 10.1371/journal.pone.0234973

**Published:** 2020-06-19

**Authors:** Sandra E. Zaeh, Margaret M. Hayes, Michelle N. Eakin, Cynthia S. Rand, Alison E. Turnbull

**Affiliations:** 1 Division of Pulmonary and Critical Care Medicine, Johns Hopkins University School of Medicine, Baltimore, Maryland, United States of America; 2 Division of Pulmonary and Critical Care Medicine, Beth Israel Deaconess Medical Center, Harvard Medical School, Boston, Massachusetts, United States of America; 3 Shapiro Institute for Education and Research at Harvard Medical School and Beth Israel Deaconess Medical Center, Boston, Massachusetts, United States of America; 4 Outcomes After Critical Illness and Surgery (OACIS) Group, Johns Hopkins University, Baltimore, Maryland, United States of America; 5 Department of Epidemiology, Johns Hopkins Bloomberg School of Public Health, Baltimore, Maryland, United States of America; University of Auckland, NEW ZEALAND

## Abstract

**Background:**

On-line tutorials are being increasingly used in medical education, including in teaching housestaff skills regarding end of life care. Recently an on-line tutorial incorporating interactive clinical vignettes and communication skills was used to prepare housestaff at Johns Hopkins Hospital to use the Maryland Orders for Life Sustaining Treatment (MOLST) form, which documents patient preferences regarding end of life care. 40% of housestaff who viewed the module felt less than comfortable discussing choices on the MOLST with patients. We sought to understand factors beyond knowledge that contributed to housestaff discomfort in MOLST discussions despite successfully completing an on-line tutorial.

**Methods:**

We conducted semi-structured telephone interviews with 18 housestaff who completed the on-line MOLST training module. Housestaff participants demonstrated good knowledge of legal and regulatory issues related to the MOLST compared to their peers, but reported feeling less than comfortable discussing the MOLST with patients. Transcripts of interviews were coded using thematic analysis to describe barriers to using the MOLST and suggestions for improving housestaff education about end of life care discussions.

**Results:**

Qualitative analysis showed three major factors contributing to lack of housestaff comfort completing the MOLST form: [1] physician barriers to completion of the MOLST, [2] perceived patient barriers to completion of the MOLST, and [3] design characteristics of the MOLST form. Housestaff recommended a number of adaptations for improvement, including in-person training to improve their skills conducting conversations regarding end of life preferences with patients.

**Conclusions:**

Some housestaff who scored highly on knowledge tests after completing a formal on-line curriculum on the MOLST form reported barriers to using a mandated form despite receiving training. On-line modules may be insufficient for teaching communication skills to housestaff. Additional training opportunities including in-person training mechanisms should be incorporated into housestaff communication skills training related to end of life care.

## Introduction

The Maryland Medical Orders for Life Sustaining Treatment (MOLST) form creates portable and enduring medical orders documenting patient preferences regarding cardiopulmonary resuscitation (CPR) and life-sustaining treatments such as mechanical ventilation.[1] The Maryland MOLST is similar to the Physician Orders for Life-Sustaining Treatment (POLST) form that is currently used in 46 states, but differs in that completing the Maryland MOLST is mandatory for adult patients being discharged to a nursing home, assisted living facility, home health agency, hospice, dialysis center, or being transferred between healthcare facilities.[2][3] Physicians, nurse practitioners, or physician assistants complete MOLST paperwork documenting a patient’s preferences for life sustaining treatment after discussion with the patient or the patient’s health care agent. The first page of the MOLST, which asks about a patient’s resuscitation status, must be filled out for these patients, while the second page, which focuses on situations other than cardiopulmonary arrest, is optional. Facilities and EMS providers can use MOLST forms to guide the use or withholding of life sustaining treatment.

In 2013, the state of Maryland decided that MOLST forms could be created and signed by trainee physicians (housestaff),[4] leading to the challenge of ensuring that housestaff were prepared to engage in conversations with patients regarding their preferences about end of life care. Prior research has demonstrated that housestaff feel both unprepared and uncomfortable talking to patients about end-of-life care despite being responsible for conducting and documenting conversations with terminally ill patients.[5][6]

A number of previously created curricula have improved housestaff communication skills and comfort with conducting code status discussions by including multimodal methodologies such as simulation, the use of lectures and clinical vignettes, small group practice, and on-line modules.[7][8][9] On-line tutorials are increasingly used in medical education,[10] and have been used as a form of continuing medical education for health care employees for the purpose of dissemination of information such as hospital policies.[11] They have been shown to be effective when compared to instructor led workshops and written training modules.[12]

Existing communication skills curricula for housestaff are time and resource intensive and do not contain state-specific information related to the MOLST. Therefore, to help housestaff understand and feel more clinically competent and comfortable completing MOLST forms, Johns Hopkins Hospital created a voluntary, on-line educational module incorporating interactive clinical vignettes and communication skills demonstrations. An assessment conducted two months after module completion found the module had no effect on trainee knowledge.[13] Furthermore, 40% of housestaff who viewed the module endorsed being less than comfortable in discussing the choices on a MOLST form with patients.[13]

The purpose of this qualitative study was to identify and understand housestaff barriers beyond knowledge that contributed to lack of comfort with the MOLST form. Our analysis suggested that such factors included physician barriers to completion of the MOLST (i.e. lack of experience and emotional fatigue), perceived patient barriers (i.e. lack of patient understanding and timing of MOLST conversations), and characteristics of the MOLST form.

## Methods

### Participants

Participants were housestaff at Johns Hopkins Hospital who completed a pre/post evaluation and an on-line training module about the MOLST form. Housestaff knowledge regarding which patients require completed MOLST forms at discharge, which health-care workers are authorized to sign MOLST forms, and how MOLST forms and advance directives differ conceptually and legally were assessed on a scale of 0 to 23.[13] Housestaff also rated their comfort discussing the choices on a MOLST form with patients using a 5-point Likert scale ranging from very comfortable to very uncomfortable as part of the post-intervention assessment.

358 housestaff completed the on-line module and pre/post evaluation with a mean knowledge score of 16 on the post-intervention assessment. The 32 housestaff who scored above the mean (>16) on the knowledge assessment, but described themselves as very uncomfortable, uncomfortable, or neutral discussing choices on the MOLST form were eligible for follow-up interviews. 7 housestaff reported they had never completed a MOLST form or were housestaff in subspecialties that do not routinely discharge patients requiring MOLST forms (i.e. emergency medicine), making them ineligible. A total of 23 eligible housestaff received an e-mail invitation to participate in a 30 minute, one-on-one, telephone interview (2 housestaff had left Johns Hopkins). 19 (83%) eligible housestaff responded to the interview request, and 18 (78%) completed their interviews within five weeks of the invitation. Interviews lasted for an average duration of 22 minutes. All housestaff who responded to the e-mail invitation provided oral informed consent prior to the start of the interview. Housestaff received a $50 Amazon.com gift card for their participation. This study and the oral informed consent process was approved by the Johns Hopkins University Institutional Review Board (IRB). The interviewer reviewed and documented oral informed consent prior to the initiation of the interview.

### Training module

The on-line training module included: (1) a video introduction by a former Johns Hopkins chief medical resident (author MMH), (2) a video called “Palliative Care and the Human Connection: Ten Steps for What To Say and Do,” featuring Diane Meier (Director, Center to Advance Palliative Care, NY)” (3) a video regarding Advance Care Planning Decisions titled “Goals of Care: A General Overview” (2008–2013 Nous Foundation, MA), and (4) interactive case scenarios focusing on how to complete and interpret MOLST forms, emphasizing the differences between the MOLST and Advance Directives.[13]

The module was available to all employees of the academic medical center and required 25 to 30 minutes to view. It was possible for viewers to skip the videos in the module but not the interactive cases. Viewers were required to select correct responses to questions related to the cases to proceed.

### Interviews

Semi-structured telephone interviews were conducted during September 2015 using a written interview guide. The interview guide, created by the authors via an iterative process, asked housestaff about their experiences with completing the MOLST form and their comfort discussing treatment options on the MOLST form with patients. It was revised based on three pilot interviews with housestaff who were not eligible to participate prior to study recruitment. The interview guide included open-ended questions about housestaff educational experiences with the MOLST form and about housestaff comfort completing MOLST forms with patients. All interviews were conducted by a single researcher (SEZ), audiotaped, and transcribed by a professional medical transcriptionist. The interviewer had training in qualitative methodology and was also a member of the housestaff at the time of the interviews. Interviewees were informed that the interviewer was a member of the housestaff interested in understanding housestaff experiences with the MOLST form, and the interview would remain anonymous.

### Analysis

Transcripts were analyzed using NVivo 10.0 software (QSR International Pty Ltd, 2013, Doncaster, Australia). Two investigators (SEZ, MMH) independently read all transcripts and performed line by line inductive coding of the transcripts, allowing for direct generation of themes from interviewee comments. The two coders met with senior researcher (AT) to review the themes that had emerged and to develop a codebook that was used to guide transcript analysis for all interviews. The two coders continued to code using an iterative process, meeting after every 5 interviews to again discuss emerging themes and assess any discrepancies. Thematic saturation was reached at 13 interviews, with no novel themes emerging over the course of three consecutive interviews, but interviews with all eligible and interested participants continued to be conducted. With the assistance of a qualitative expert who was not involved in data collection (ME), thematic analysis was conducted to categorize themes into meaningful concepts for presentation of results.

## Results

Among the 18 housestaff who were interviewed, 6 (33%) reported neutral comfort, 3 (17%) reported they were uncomfortable, and 9 (50%) said they were very uncomfortable. Five interviewees (28%) had completed their intern year, 7 (39%) interviewees were second year residents, and 6 (33%) interviewees had completed at least two years of training. Nine (50%) of the participants were from the Department of Internal Medicine. More than a third of interviewees (n = 7, 39%) reported that they complete MOLST forms more than once a week, but less than daily (Table 1).

**Table 1:** Demographics of the 176 house staff with a MOLST knowledge score >16 by self-reported comfort discussing the choices on a MOLST form with patients.

*“How comfortable do you feel discussing the choices on a MOLST form with patients*?*”* →	Comfortable or very comfortable (N = 144)	Neutral, Uncomfortable, or Very Uncomfortable (N = 32)	Interviewed^1^ (N = 18)
**Post-graduate training year, N (%)**			
Intern year completed	42 (29%)	6 (19%)	5 (28%)
2nd year completed	47 (33%)	9 (28%)	7 (39%)
≥ 3rd year completed	55 (38%)	17 (53%)	6 (33%)
**Post-graduate training program, N (%)**^2^			
Internal Medicine	60 (42%)	10 (31%)	9 (50%)
General Surgery	21 (15%)	4 (13%)	2 (11%)
Surgical sub-specialties	15 (10%)	7 (22%)	2 (11%)
Gynecology & Obstetrics	14 (10%)	4 (13%)	4 (22%)
Neurology	13 (9%)	0 (0%)	0 (0%)
Emergency Medicine	8 (6%)	4 (13%)	0 (0%)
Psychiatry	8 (6%)	3 (9%)	1 (6%)
Physical Medicine & Rehabilitation	5 (3%)	0 (0%)	0 (0%)
**How frequently do you complete Maryland Orders for Life Sustaining Treatment (MOLST) forms with patients?**^2^ **N (%)**			
Never	5 (3%)	4 (13%)	0 (0%)
Less than once a month	36 (25%)	10 (31%)	4 (22%)
>once a month but <once a week	24 (17%)	5 (16%)	3 (17%)
> once a week but <daily	66 (46%)	9 (28%)	7 (39%)
At least once each day	13 (9%)	4 (13%)	4 (22%)
**Who** **primarily** **coordinates discharges on your floor/service?**^2^ **N (%)**			
House staff	115 (80%)	23 (72%)	14 (78%)
NPs, PAs, SWs, Discharge Planners or Other	29 (20%)	9 (28%)	4 (22%)
**How confident are you in your ability to properly complete a MOLST form with patients?**^2^ **N (%)**			
No or minimal confidence	1 (1%)	2 (6%)	0 (0%)
Moderate confidence	17 (12%)	14 (44%)	6 (33%)
Good or complete confidence	126 (88%)	16 (50%)	12 (66%)

**Abbreviations**: NP, Nurse Practitioner; PA, Physician Assistant; SW, Social Worker.

^1^Only house staff with a test score >16 who reported that they were Neutral, Uncomfortable, or Very Uncomfortable discussing the choices on a MOLST form with patients were eligible for interview. Emergency medicine residents were ineligible because they do not routinely discharge patients. One general surgery resident and 2 surgical subspecialty residents were ineligible because they reported never having completed a MOLST form.

^2^Percentages may not sum to 100% due to rounding.

The coding performed by the two coders showed good agreement (percent agreement = 98%) and substantial inter-rater reliability (Cohen’s K = .74). Qualitative analysis showed that there were three major factors contributing to lack of housestaff comfort completing the MOLST form with patients: (1) physician barriers to completion of the MOLST, (2) perceived patient barriers to completion of the MOLST, and (3) design characteristics of the MOLST form. In addition to these three themes, a fourth theme of (4) adaptations for improvement was identified.

### Physician barriers to comfort in completion of MOLST

Interviewees expressed several physician-related barriers to comfort in completing the MOLST form with patients, starting with lack of experience (Table 2). Despite the fact all interviewees completed the on-line module, housestaff reported they had received minimal training on proper MOLST form completion and lacked knowledge of which parts of the form needed to be completed. Several housestaff reported that their comfort with form completion increased with time over the course of their medical training. As one participant described, “When I first started as an intern it was extremely painful because it was unclear what you had to do or who was supposed to do it, but now it seems relatively straightforward.” A lack of experience was also endorsed by physicians practicing in specialties where the MOLST form was completed infrequently. As an OB/GYN resident said, “I haven’t had a great deal of experience talking about end of life issues, because it only comes up in certain subfields of OB/GYN. It doesn’t come up in the majority of my rotations.”

**Table 2:** Physician barriers to comfort in completion of MOLST.

Theme	Sub-Theme	Exemplar Quote
Lack of experience	Difficult at the beginning of training	“Early on in the year I didn’t really understand which patients needed it and which didn’t and had some confusion about how much of the MOLST needed to be filled out. I think at the end of the year I got a little better at understanding who needed it.”
	Lack of training	“So I feel like there wasn’t much training. I remember randomly intern year we were supposed to start doing them.”
Emotional Fatigue		“I can’t remember any positive experiences [discussing the MOLST with patients] because it isn’t something that I particularly like talking about…when we’re talking about end of life decisions, we’re talking about this worst case scenario type of situation and I’m in a field where most of my patients are healthy and so talking about this brings up the question, well why are you thinking about this?”
Overstepping role	Role of attending physician	“I do remember there was a particular attending who felt like it was a very important thing that they wanted to do, conversation that they wanted to have themselves.”
	Role of outpatient provider	“I think these conversations are best carried out by the primary care physician, you know because it is part of the overall part of being a patient and overall health of a patient, so I think that’s best managed by a primary care doctor.”

An additional physician barrier was emotional fatigue in discussing patient’s preferences about end of life care. As one participant stated, “I think it’s part of our job but I hate being the person to bring bad news and I hate being the one to break the news…it feels sad and emotionally draining and the conversation can be tiring.”

Several housestaff felt they were “overstepping” their role by completing the MOLST form with patients. Housestaff, primarily in surgical specialties, believed that discussing end of life preferences with a patient was an important conversation that should involve the attending physician. As an OB/GYN resident said, “That’s an attending level conversation and I would be overstepping my position if I were to bring that up out of the blue.” A general surgery resident described the role of being a resident as “awkward in the sense that you may have an opinion about something that may not be an opinion your attending shares.”

Other housestaff felt that by discussing end of life care preferences with patients admitted to the hospital, they were overstepping the role of outpatient providers, who had a long term relationship with the patient and better understanding of the patient’s clinical course. As one participant said, “I think patients view it …as less of an imposition or an overstepping when their longitudinal physician says, I know you, you know me, we’ve been working together and trying to maximize your quality of life and part of that means if a bad thing were to happen, what would you want.”

### Perceived patient barriers leading to lack of physician comfort

Participants described a number of perceived patient-related barriers, also contributing to lack of physician comfort in completing the MOLST (Table 3). Interviewees explained that some patients did not understand the purpose of MOLST completion. Patients who had advance directives in place were not eager to complete MOLST forms as they did not comprehend how the MOLST “would modify anything.” Housestaff were not always able to fully explain the differences between types of legal paperwork to patients. Other interviewees encountered patients who believed that discussion of MOLST preferences suggested the patient had a poor prognosis, rather than serving as a mechanism to document a patient’s preferences for future care. As one participant said, “I had some very awkward experiences with patients where they didn’t understand the point of the form and one person had a mini anxiety attack because I was asking her all of these questions and she thought she was going to die.” Several patients were felt to be “too healthy” to require a mandatory MOLST form, making it difficult to comfortably engage in a conversation about code status. As an interviewee said, “It’s obviously very important for patients who are becoming terminal, but for patients who are otherwise healthy, approaching that subject feels awkward, like I don’t know how to preface the conversation in a way that is appropriate.”

**Table 3:** Perceived patient barriers leading to lack of physician comfort.

Theme	Sub-Theme	Exemplar Quote
Lack of patient comprehension	MOLST vs. advance directives	“One patient had filled out an entire advance directive and all these other legal forms and wanted to know exactly how this form came into the picture and how does it modify anything, and I don’t think I exactly had the answer to that.”
	Concern for poor prognosis or medical team will not act	“I have had some instances where a patient will say, well why are you asking me this, and they get scared that we aren’t going to do everything…"
	Patient considered to be "too healthy" to complete MOLST	“I have to do the MOLST as a mandatory component of discharge and I’m going through it with a patient and they’re not really someone who has true sick potential in this acute situation and it opens Pandora’s box about transfusions and dialysis and in that situation, it’s like, did we really need to have this conversation? They are here for cellulitis that didn’t need to be admitted and they need home care.”
Timing of MOLST conversation	Challenging to perform at time of discharge	“People are always confused as to why they need to answer these questions because most of the time we’re filling them out at the end of their stay, and they are fine, and all of a sudden you’re asking them about goals of care.”
	Requesting more time to consider wishes	“Some people say I don’t know, I can’t make that decision right now. There is a box where you can plead that, but it pretty much means they would be resuscitated.”

Interviewees perceived patient frustrations related to the timing of MOLST completion. As the MOLST is typically filled out just prior to discharge, housestaff perceived that the timing felt “out of left field” for patients who had experienced clinical improvement over the course of their hospitalization. As a participant described: “They had a long hospitalization, it was emotionally challenging for them, and now congratulations, you are looking better, you get to go home, and by the way you need a MOLST form filled out and you need to tell us what you would want us to do if you died.” Other patients requested additional time to consider their wishes, rather than being rushed to do so on the day of discharge.

### Design characteristics of MOLST forms contributing to physician discomfort

Housestaff discussed design characteristics of the MOLST form that contributed to physician discomfort (Table 4). The MOLST was described as being similar to ‘a checklist’ with a participant stating that completion of the MOLST was “more of a task of just completing it as paperwork and less of a discussion.” While several interviewees stated they believed the intent of the MOLST form was to encourage physicians to have conversations about a patient’s preferences for care at the end of his or her life, many described it as an ineffective or “artificial” tool that did not help facilitate those conversations. As one interviewee said, “The MOLST doesn’t facilitate the most wonderful goals of care discussions…it feels very, very removed from the patient’s involvement in their health care and I think that’s the opposite of what it’s trying to achieve.”

**Table 4:** Characteristics of MOLST leading to lack of physician comfort.

Theme	Exemplar Quote
Task of Paperwork	“It’s become much more run of the mill for me and now it just becomes more of a task of completing it as paperwork and less of a discussion. I think probably the goal of MOLST was to have real conversations with patients about their goals of care rather than just checking off something prior to transport.”
Poor facilitator of goals of care conversations	“I care more about what the patient’s goals of care are, and I don’t really care all that much about the MOLST. The MOLST is a sheet of paper that the state of Maryland requires us to have in certain situations and independent of that, discussions still need to be had and I would still have them with my patients.”
Lack of clarity of second page of the MOLST	“I mean the code, full code/not full code stuff I’m perfectly fine with. I think the second page of the MOLST where they want specific amounts of time that you would give artificial nutrition or ventilate, I think the way that form is phrased is incredibly awkward and I’m not comfortable with it.”

Housestaff explained that the first page of the MOLST form, which asks about a patient’s resuscitation status, is relatively straightforward and easy to understand, but the second page of the MOLST, which focuses on patient’s wishes regarding subjects including artificial ventilation, blood transfusion, dialysis, and artificial nutrition (among other interventions), was confusing for patients. As a participant said, “I feel very comfortable on the first page…and that’s the big picture resuscitation thing. I feel less comfortable on things like hydration, dialysis, and feeding…because I think it’s hard to generalize those sorts of wishes.” Other housestaff described uncertainty as to when they were required to complete the second page for patients, with one participant describing it as a “mysterious page that doesn’t often get filled out.”

### Adaptations for improvement of MOLST conversations

Housestaff interviewees proposed recommendations for how to adapt medical education to improve housestaff comfort in facilitating MOLST conversations (Table 5). Several participants discussed involvement of the palliative care team in housestaff training for completion of the MOLST form. Housestaff reported that they often learned how to best approach conversations regarding end-of-life preferences by modeling their behaviors after palliative care physicians. One participant explained that they valued “watching people who are good at it [discussing end of life preferences] and picking up on how they structure the conversation, morphing that…and sort of making it your own going forwards.”

**Table 5:** Adaptations for improvement of MOLST conversations.

Theme	Sub-theme	Exemplar Quote
Involvement of palliative care team	Modeling behavior of palliative care physicians	“I think the palliative care person, it was nice to hear her go through it because they have a different take…They just have a much deeper understanding than typically we get, so it was nice to hear her go through it.”
	Involvement of palliative care in housestaff training	“We had a really good palliative care program at the medical school that I trained at, and as a medical student we actually got some pretty good foundational teaching about how to structure a goals of care conversation and an end of life conversation, and I think that’s one of the more useful things, getting a good foundation so you can have a sense of what’s important about having those conversations.”
	Have a larger role in patient care	“We’re kind of like short of time and don’t know a lot about the legal stuff, so I think it would be helpful if there was someone we could lean on when patients either weren’t sure or wanted more information or something more in depth than a MOLST.”
Alternative modes of training		“It may be helpful to have a group learning or group learning module that you go through together.”

Housestaff also recommended additional training via alternative modalities on conduction of conversations regarding end of life preferences, including group learning modules, role playing, or receiving feedback on family meetings they led. As one participant said, “…I feel that some didactic and some actual practicum would be helpful. And perhaps engaging some of the people in our community, like someone who had a family member die in the ICU. Come back and talk to us about how those decisions sounded when they came from us. Because we know what we think we are saying but…who knows if that is what they are hearing.”

## Discussion

In this qualitative study of 18 housestaff with above average knowledge regarding the MOLST form, there were a number of barriers that contributed to housestaff discomfort. Physician barriers to completion of the MOLST included lack of experience, emotional fatigue, and the feeling of “overstepping” one’s responsibilities. Perceived patient barriers leading to lack of physician comfort included lack of patient understanding regarding the purpose of the MOLST and patient frustration regarding the timing of MOLST completion. Characteristics of the MOLST, such as its format as a checklist and confusion regarding the first versus second page of the form, also contributed to resident discomfort. Housestaff felt that greater exposure to the palliative care team and additional training via role playing, direct observation and feedback as well as more patient involvement would facilitate their training.

Prior studies of the POLST have suggested it is an effective tool in ensuring treatment provided at the end of life matches the orders on the form.[14] Health care providers generally found the POLST to be helpful in guiding discussions, but a number of challenges exist with understanding the form.[15] For example, there is a lack of consistency in form interpretation by out of hospital personnel and emergency room physicians [16] and concern that the form may decrease patient centered decision making.[17] A chart review investigating use of the Maryland MOLST found contradictions between health care provider responses on page one and two of the MOLST form, in line with our study results which found physicians to be confused regarding the first and second page of the form.[18]

Our results are consistent with prior studies of resident experiences with end-of-life decision making.[5] Several studies have described resident discomfort stemming from a lack of training and support in conducting goals of care conversations.[19][20] Stevens et al. found that close to 80% of residents stated they had received none or too little support in leading end of life decision making prior to starting an ICU rotation.[20] Additional barriers to conducting goals of care conversations include lack of continuity in clinical relationships, the perception that a patient was not sick enough to have a goals of care conversation[21], personal discomfort, challenging family dynamics, and lack of time.[22] Our study is unique in its assessment of qualitative barriers to physician comfort following an on-line education module including communication skills focused on the MOLST. This is important given the increasing role of physician orders for life sustaining treatment forms nation-wide as a mechanism to document patient preferences regarding end of life care.

The barriers to comfort that exist after completion of an on-line communication skills module for knowledgeable housestaff suggest on-line modules may not be sufficient for teaching communication skills regarding goals of care conversations. Despite completing the on-line curriculum, housestaff felt they had received inadequate training on how to properly complete the MOLST and discuss patients’ preferences at the end of life. There has been previously documented success with on-line palliative care curricula, although the need for in-person multifaceted education to solidify newly learned skills from such curricula into practice was acknowledged.[23]

This study highlights the needs for effective education programs to improve end of life care training for residents. While multi-modality educational methods such as readings, on-line training, group discussions and problem based learning have been successfully used in the past to improve end of life communication skills, in-person training mechanisms, such as role playing and simulation training should also be considered for the technical aspects of end of life communication.[8][9][24] Real life practical application in supervised family meetings with opportunities for direct feedback may also be effective for developing skills.[9][24] In addition to highlighting the need for more hands on training, residents in this study felt direct observation of senior colleagues, notably palliative care specialists, performing MOLST discussions would be helpful, reflecting the importance of apprenticeship as a learning mechanism.[25] When developing communication skills, housestaff seek out concrete behaviours and sentences from role models which they then repeatedly attempt and personalize through practice.[26] Both acquisition through apprenticeship and experiential learning through repetition are essential [27] as more end-of-life discussions per ward-month are significantly associated with greater behavioral competence in conducting end of life conversations. [28]

Additionally, while interviewed housestaff had mastered the formal MOLST curriculum, many were uncertain if the curriculum was relevant to their clinical practice. In medical education the formal, or stated and endorsed curriculum, may differ substantially from the hidden curriculum, which conveys an understanding of how the medical system works in practice.[25] While the formal curriculum stated that housestaff should complete MOLST forms, implicit messaging conveyed that MOLST forms are insignificant, or serve to codify and legitimate discussions that only attending physicians may perform. The importance of the MOLST must be more clearly conveyed to housestaff. Similarly, housestaff should receive clear guidelines about their role in completing the MOLST with patients, along with training about how to best do so specifically addressing subjects confusing to housestaff, such as the role of the first versus the second page of the MOLST.

The limitations of our study include its small sample size and single center design. We did not interview housestaff who expressed that they were comfortable discussing MOLST preferences, choosing to focus on housestaff who described themselves as being less than comfortable. Furthermore, we did not formally assess clinical competence of housestaff in discussing MOLST preferences with patients, but this could be performed in the future. Due to the use of qualitative interviews with housestaff within this study, the data may be subject to self-report or recall bias. Finally, this qualitative analysis was performed after conducting our on-line training module. Future education initiatives may benefit from initial qualitative needs assessments investigating the needs of participants.

The study’s strengths include its exploration of factors beyond knowledge that contribute to housestaff discomfort in discussing patient preferences for end of life care. Elements of these findings are transferable to scenarios beyond the MOLST form and specifically could relate to how housestaff are educated regarding use of the POLST form and conducting goals of care conversations.

## Conclusions

In conclusion, some housestaff with above average performance on a test about interpretation and completion of a portable medical order form expressed lack of comfort in completing the form with patients. Factors cited as contributing to discomfort included physician barriers, perceived patient barriers, and design characteristics of the MOLST form. On-line modules may not be sufficient for teaching communication skills to housestaff in discussing end of life care with patients. Additional training opportunities including in-person training mechanisms should be incorporated into housestaff communication skills training related to end of life care.

## Supporting information

S1 Appendix Codebook. (DOCX)
