# Supplementary material for: Housestaff perceptions on training and discussing the Maryland Orders for Life Sustaining Treatment Form (MOLST)
Source: PLoS One. 2020 Jun 19;15(6):e0234973. doi: 10.1371/journal.pone.0234973 (PMC7304571; doi:10.1371/journal.pone.0234973)
Supplement: S1 Appendix — (DOCX) [file pone.0234973.s001.docx]

**S1. Appendix: Codebook**

| **Code** | **Definition of Code** |
| --- | --- |
| Admission code discussions | Residents describing having or not having code discussions on admission. |
| Advanced directives | Advanced directives are discussed in comparison to the MOLST. |
| Annoying | “Annoying,” “annoy,” “annoyance” mentioned. |
| Attendings | Attending physicians are mentioned. |
| Awkward | Interviewee describes MOLST process as being “awkward.” |
| Bad News | Providing or giving “bad news” described. |
| Cancer | Cancer or oncology is mentioned. |
| Case manager or social worker | Case manager or social worker referenced. |
| Challenges with end of life discussions | Interviewee describes challenging aspects of end of life discussions. |
| Changes in code status | Situation where patients change code status is discussed. |
| Checking off a list | Filling out the MOLST is described as checking off a list. |
| Chief resident | Chief resident is mentioned. |
| Code discussion other than completing a MOLST | Description of code discussions separate from MOLST form completion. |
| Code Status | Code status: Full Code, DNR/DNI, Limit, Full. |
| Comfort | Interviewee expresses comfort discussing MOLST with patients. |
| Comfort change | Resident describes how comfort level with completing the MOLST has changed over time. |
| Comparison | Resident compares code discussions with and without use of the MOLST. |
| Completion of MOLST | Description of how the MOLST is completed with a patient. |
| Confidence | Resident describes confidence in completing MOLST. |
| CPR | Use of “CPR” described. |
| Decision maker | Mention of a decision maker or surrogate. |
| Decisions broader than end of life decisions | Reference to decisions patient or surrogate needs to make that are more general than end of life decisions. |
| Dialysis | Dialysis described in text. |
| Different than TV | Resident compares code as being different than what occurs on television. |
| Discharge | MOLST is brought up at the time of discharge. |
| Discomfort | Resident describes discomfort in completing MOLST with patient. |
| Distaste | Expresses distaste or dislike of the MOLST form. |
| Does not review MOLST with Patient | Resident does not review the MOLST with patients. |
| Education | How resident learned how to complete the MOLST. |
| Emotional | End of life or MOLST conversation is described as “emotional.” |
| End of life decisions | “End of life decisions” are discussed. |
| First versus second page of MOLST | Specific reference to the first versus the second page of the MOLST. |
| Frustration | Interviewee describes patient frustration with the MOLST form. |
| Goals of care | The term “goals of care” is mentioned. |
| Helpful language | Resident describes helpful language used when explaining MOLST form to patients. |
| Hope | Concept of hope is mentioned by the resident. |
| ICU | The Intensive Care Unit is referenced by the physician. |
| Ineptitude | Lack of skill or ability in completing the MOLST form is described by the resident. |
| Inpatient | Resident discusses preference for completing MOLST in the inpatient setting. |
| Intern | Interns are mentioned in discussing the MOLST. |
| Lack of understanding | Resident describes a lack of understanding of the MOLST on the part of the patient or the physician. |
| Legal | Legal questions regarding the MOLST. |
| Modeling | Modeling behavior is described as possible training mechanism |
| Need for MOLST | Resident describes his or her thoughts on whether there is a need for the MOLST form. |
| Negative Experience | Negative experiences related to the MOLST are described. |
| Noninvasive ventilation | CPAP or BiPap are mentioned. |
| Options or choices on the MOLST form are discussed | Options or choices on the MOLST form are discussed. |
| Outpatient discussion | Reference to outpatient discussion regarding completion of the MOLST form. |
| Overstepping | Residents describe feeling they are “overstepping” their role by completing the MOLST. |
| Palliative care | Palliative care team involvement is described. |
| Paperwork | MOLST is described as “paperwork.” |
| Part of the job | Filling out MOLST described as part of one’s job. |
| Patient preference | Patient preference regarding goals of care or wishes is discussed. |
| Patients unsure | Patients are unsure of how they should complete their MOLST form. |
| Positive experience | Residents describe a positive experience related to the MOLST. |
| Quality of Life | Quality of life is discussed. |
| Real goal of the MOLST | Residents refer to what they believe the “real goal” of the MOLST form is. |
| Redesign | Interviewee describes how they would redesign the MOLST. |
| Relationship | Reference to the patient-doctor relationship. |
| Responsibility for establishing goals of care | Residents discuss whose responsibility it is to establish goals of care of the patient. |
| Responsibility for completing MOLST forms | Discussion of whose responsibility it is to complete MOLST forms. |
| Time | Residents describe length of time to complete MOLST form. |
| Training | Future training initiatives that should occur for MOLST education are described. |
| Uncomfortable | Interviewee describes lack of comfort completing the MOLST form with the patient. |
| Video Training Module | Video training module suggested as possible educational technique for residents. |
| Without MOLST | Residents describe goals of care discussions being easier without the MOLST. |
